# Genetic insights into lipid traits and atherosclerosis risk: a Mendelian randomization and polygenic risk score analysis

**DOI:** 10.1097/JS9.0000000000002869

**Published:** 2025-08-12

**Authors:** Hongliang Zhang, Xiaoyu Long, Guannan Niu, Wence Shi, Zhenyan Zhao, Dejing Feng, Hui Sun, Yongjian Wu

**Affiliations:** a Department of Cardiology, Fuwai Hospital, National Center for Cardiovascular Diseases, Chinese Academy of Medical Sciences and Peking Union Medical College, Beijing, China; b Department of Endocrine, Seventh Medical Center of Chinese PLA General Hospital, Beijing, China

**Keywords:** atherosclerosis, drug targets, lipid traits, Mendelian randomization, polygenic risk score

## Abstract

**Background:**

Atherosclerosis (AS) is a leading cause of cardiovascular diseases, with lipid metabolism disorders playing a key role in its development. This study used Mendelian randomization (MR) analysis to examine the causal links between four lipid traits [high-density lipoprotein (HDL), low-density lipoprotein (LDL), triglycerides (TG), and total cholesterol (TC)] and AS risk, and also investigated the polygenic risk score (PRS) and potential molecular mechanisms.

**Methods:**

Genome-Wide Association Study GWAS summary data for lipid traits from the Integrative Epidemiology Unit IEU and Finngen databases were used for MR analysis to assess the causal link between lipid traits and AS risk. The odds ratios (ORs) and 95% confidence intervals (CIs) were calculated. Single-cell RNA sequencing (scRNA-seq) and bulk RNA sequencing (bulk RNA-seq) data were used to evaluate the PRS of AS. Drug enrichment analysis and molecular docking were performed to identify potential drug targets.

**Results:**

Higher HDL levels were associated with a decreased risk of AS (OR = 0.8038, *P* = 0.000014), while higher LDL, TC, and TG levels were linked to increased AS risk (OR = 1.0147, *P* = 9.95 × 10^−13^; OR = 1.0163, *P* = 8.98 × 10^−16^; OR = 1.0087, *P* = 4.32 × 10^−4^). Drug enrichment analysis highlighted potential drug targets, including HMGCR binding with STIGMASTEROL and Benzofurans. PRS analysis revealed that multiple lipid metabolism-related genes influence AS susceptibility.

**Conclusion:**

The study demonstrated a clear causal relationship between lipid traits and AS risk. Higher HDL levels were associated with a reduced risk, while higher LDL, TC, and TG levels increase AS risk. The role of lipid metabolism genes in AS pathogenesis was underscored by PRS analysis.

## Introduction

Atherosclerosis (AS) is a critical pathological basis for cardiovascular diseases worldwide, and research indicates that abnormal lipid metabolism plays a key role in its pathogenesis^[1,2]^. Changes in lipid traits, such as high-density lipoprotein (HDL)^[3]^, low-density lipoprotein (LDL)^[4]^, triglycerides (TG)^[5]^, and total cholesterol (TC)^[6]^, are closely associated with the development and progression of AS. Despite these associations, the causal relationship between lipid traits and AS remains a topic of debate. Traditional observational studies are prone to confounding factors, making it challenging to determine whether these lipid abnormalities are directly driven by AS or merely correlated biomarkers of increased cardiovascular risk. This uncertainty highlights the need for further research, especially using methods like Mendelian randomization (MR), to clarify these relationships and establish definitive causal links.

**HIGHLIGHTS**

- reveal multiple lipid metabolism-related genes influence AS susceptibility
- indicate decreased levels of HDL and elevated levels of LDL, TC, and TG significantly increased the risk of AS
- demonstrate a clear causal relationship between lipid traits and AS risk
- underscore the relevance of genetic insights in understanding and managing AS and related cardiovascular conditions.

Recently, MR has been widely used to explore the causal links between lipid traits and diseases. Through this method, researchers can identify potential drug target genes based on genetic variation and assess their roles in lipid metabolism and atherosclerotic cardiovascular disease (ASCVD) risk. This process not only complements traditional clinical trials and observational studies but also provides crucial evidence for the development of new therapeutic strategies. For instance, studies have shown that certain lipid-lowering drugs exhibit strong causal effects on lowering cholesterol levels through the genetic variations of their target genes. By conducting MR analyses of drug targets, it is possible to identify genes related to lipid metabolism, thereby providing precise targets for the development of next-generation lipid-lowering drugs^[7–9]^. Moreover, the relationship between lipid traits and ASCVD is not the result of a single factor but rather the outcome of interactions among multiple genes. Consequently, constructing polygenic risk scores (PRSs) as an effective means to assess individual disease risk has gradually attracted attention from researchers^[10,11]^. By integrating information on numerous genetic variants associated with lipid traits, PRS can comprehensively evaluate an individual’s genetic susceptibility across multiple gene loci, thus predicting the risk of AS. In multivariable Mendelian randomization (MVMR) analysis, combining genetic data from multiple lipid traits enables a more precise evaluation of their individual and combined effects on AS. It also helps clarify how different lipid components interact through various biological mechanisms to affect AS onset and progression.

This study collected summary-level genome exhaustive association study (GWAS) data for four lipid traits (HDL, LDL, TG, and TC) from the IEU and Finngen databases. It assessed the causal relationship between lipid traits and ASCVD risk through two-sample MR analysis, identifying potential drug targets. Drug enrichment analysis revealed significant associations between 3-Hydroxy-3-Methylglutaryl-CoA reductase Gene (HMGCR) and 2,4-Dibromophenol, 3,3’,4,4’-Tetrabromobiphenyl, 3,3’,4,4’,5,5’-Hexabromobiphenyl, Benzofurans, and Stigmasterol. Molecular docking analysis showed that HMGCR has strong binding affinity with both Stigmasterol and Benzofurans. Additionally, by integrating scRNA-seq and bulk RNA-seq data, we explored multi-gene risk scores and potential molecular mechanisms of AS, providing new insights for the prevention and treatment of AS.

To ensure the transparency and reproducibility of our research, particularly in the context of using advanced analytical methods and tools, we adhered to the TITAN Guidelines for reporting the use of artificial intelligence in scientific research^[12]^. Although the AI tool provided support in text generation, all content generated or influenced by AI is the responsibility of the human authors. The authors take responsibility for the integrity of the content affected/generated^[12]^.

## Methods

### Data acquisition

For this study, GWAS summary data for four lipid traits (HDL, LDL, TG, and TC) were obtained from the IEU database (https://gwas.mrcieu.ac.uk/) as exposure variables, with specific dataset IDs being “ieu-a-299,” “ieu-a-300,” “ieu-a-302,” and “ebi-a-GCST90025953.” Additionally, two sets of GWAS summary data for AS were collected as outcome variables. One set was derived from the Finnish database (https://www.finngen.fi/en/access_results) with cohort ID “summary_stats_finngen_R11_I9_CORATHER,” which includes 378 019 single-nucleotide polymorphisms (SNPs) from 56 685 samples. The other set came from the IEU database with dataset ID “ieu-a-299.” The “summary_stats_finngen_R11_I9_CORATHER” cohort was used as the discovery cohort, while the “ukb-d-I9_CORATHER” cohort was the replication cohort.

On another front, scRNA-seq data for whole calcified atherosclerotic plaques and matched proximal carotid tissue segments from six patients who underwent carotid endarterectomy were obtained from the Gene Expression Omnibus database under dataset GSE159677. Bulk RNA-seq data from 10 healthy control samples and 9 AS samples were also collected from the GSE57691 dataset to validate the diagnostic significance of key genes.

### Instrumental variable selection

First, SNPs significantly associated with HDL, LDL, TG, and TC (*P* < 5e-8) were selected. Further filtering was conducted to retain SNPs meeting the linkage disequilibrium clumping threshold of *r*^2 < 0.001 and a physical distance threshold of 10 000 kb. After retaining instrumental variables with F statistics > 10, the effect allele directions were harmonized, and strand bias issues were resolved.

### Univariate MR analysis

We utilized the “TwoSampleMR” software package^[13]^ and performed a two-sample MR analysis to separately evaluate the relationships between four lipid traits and AS outcomes. The causal relationship between HDL-C levels and the outcome was assessed using inverse variance weighted (IVW) regression and MR-Egger regression. These methods calculated causal effects and generated odds ratios (ORs) with 95% confidence intervals (CIs) for binary outcomes. Sensitivity analyses included heterogeneity testing (Cochran’s *Q* statistic), horizontal pleiotropy testing (MR-Egger intercept), outlier detection (MR-PRESSO), and leave-one-out analysis by sequentially excluding SNPs. Additionally, separate MR analyses were conducted to examine the causal relationship between genetic instrumental variables for AS and lipid traits.

### Multivariable MR analysis

We evaluated the potential causal relationship between lipid traits and the risk of AS using MVMR methods. Exposure and outcome data were extracted online using the “TwoSampleMR” and “MendelianRandomization” packages. The validity of the instrumental variables was then verified by analyzing the strength of exposure SNPs and checking for horizontal pleiotropy. We used methods like IVW, MR-Egger, and median estimation to calculate causal effects and conducted sensitivity analyses, including heterogeneity and horizontal pleiotropy testing.

### Drug target MR analysis

This study collected common lipid-lowering drugs recommended in current guidelines for dyslipidemia management, including statins, cholesterol absorption inhibitors, and proprotein convertase subtilisin/kexin type 9 inhibitors. Using the DrugBank database (https://go.drugbank.com/), we identified 20 genes encoding the pharmacological targets of these drugs. Similar to the instrumental variable selection described in Section 2.2, genetic variations within ± 200 kb of the respective genes were selected as instrumental variables. Subsequently, exposure and outcome data were formatted and harmonized using the harmonise_data(·) function to ensure consistency in causal direction. In MR analyses, we assessed causal associations between instrumental variables and outcomes using methods like IVW and MR-Egger. The robustness was evaluated with Cochran’s Q and MR-Egger intercept tests. To further validate the results, MR-PRESSO bias detection was performed to identify potential outliers, and OR values and their 95% CIs were calculated to quantify causal effects.

### Colocalization analysis

Colocalization analysis was performed within a Bayesian framework using the “coloc” package^[14]^ to assess the possibility of shared causal variants for two traits (such as gene expression and disease phenotype) within a target region. After filtering, SNPs with significant colocalization signals (SNP.PP.H4 > 0.1) were extracted. The results were interpreted based on colocalization models, evaluating whether there is a shared causal locus (H4) or independent causal variants (H3). This provides references for functional studies and causal inference.

### SMR analysis

Summary-data-based Mendelian randomization (SMR) analysis was used to identify genes causally related to the risk of AS^[15]^. Specifically, eQTL data for coronary artery tissue were downloaded from the SMR Software website (https://yanglab.westlake.edu.cn/data/SMR/GTEx_V8_cis_eqtl_summary.html). The HEIDI test was used to check whether the causal gene is due to a shared genetic variant rather than linkage disequilibrium. The SMR software was used to analyze the significance of *P*-values of different genes and the *P*-values from the HEIDI test. Genes with a significance P-value less than 0.05 and a P-value greater than 0.05 in the HEIDI test were retained for subsequent analysis.

### Molecular docking

Based on the interaction data between drugs and genes from the DSigDB database (http://dsigdb.tanlab.org/DSigDBv1.0/), we performed drug enrichment analysis using the “clusterProfiler” package. Drugs with an adjusted P-value less than 0.05 were identified as drugs related to target genes. The 2D molecular structures of small molecule drugs in mol2 format were downloaded from the PubChem database. The protein structure files (in pdb format) for the target genes were obtained from Uniprot. ChemBio 3D was then used for energy minimization calculations and exports. Water molecules were removed from the protein receptor using PyMOL software. The protein was then converted into pdbqt format in AutoDockTools-1.5.7, and GridBox parameters were set according to the ligand’s active binding site. The docking process was conducted using AutoDock Vina 1.2.3, and the results were exported. Finally, PyMOL software was used to visualize the 3D structure and identify the docking result with the strongest binding affinity.

### Analysis of scRNA-seq data

The analysis of scRNA-seq data in this study was conducted using the Seurat R package^[15]^. Data preprocessing involved creating a Seurat object from raw count data and filtering cells based on quality control metrics, including RNA feature counts, percentage of mitochondrial RNA, and percentage of ribosomal RNA. Data normalization was performed using the LogNormalize method, and 2000 highly variable genes were identified for subsequent analysis. Principal component analysis (PCA) was performed using these highly variable genes, and t-SNE was used to visualize the dimensionality reduction results. Batch effects were adjusted using the Harmony algorithm^[16]^. Subsequent clustering analysis was conducted with the Louvain algorithm, and the resolution parameter was fine-tuned according to the dataset size. Differential expression analysis was used to identify marker genes for each cluster, with a minimum log2 fold change threshold of 0.25. Cell type annotation was performed using the SingleR package with the Human Primary Cell Atlas as a reference, followed by visualization of the annotated results in a low-dimensional space and manual correction of cell types to improve accuracy^[16]^.

Gene co-expression network analysis of the scRNA-seq data was performed using the hdWGCNA method^[17]^. Initially, the Seurat object was loaded and preprocessed. Metacells were then constructed based on cell type and sample, with normalization of their expression matrix. Subsequently, interested cell types were selected, and network construction parameters (soft threshold = 8) were determined through soft-thresholding analysis, leading to the construction of a weighted gene co-expression network. Modules were delineated, and module eigengenes (MEs) were computed to elucidate the relationships between modules and samples or cell types. Eigengene-based connectivity values were computed for module-specific genes, and key hub genes within the modules were extracted.

To explore the functional characteristics and potential mechanisms of specific gene sets in monocytes, we first extracted subpopulations of monocytes and defined gene set scores. We intersected the genes identified by SMR analysis^[18]^ with those in the monocyte cell cluster module, and subsequently scored the gene sets using the AddModuleScore(·) function. The scoring results were visualized as violin plots and cell clustering heatmaps to demonstrate the distribution of gene set expression levels. Furthermore, monocyte cell populations were stratified into high-expression and low-expression cohorts based on gene scores. Differential expression analysis was then performed to identify significantly different genes between these cohorts. These differentially expressed genes were sorted by log2 fold change, and gene set enrichment analysis (GSEA) was performed using the fgsea method, with particular attention paid to significantly enriched Kyoto Encyclopedia of Genes and Genomes pathways. The top-ranked pathways were extracted for visualization.

Finally, transcription factor (TF) activity analysis in monocytes was performed by integrating the “Dorothea” package with Viper tools to elucidate differences between high-risk and low-risk groups. Initially, monocytes were filtered based on single-cell transcriptome data and redefined as high-risk and low-risk groups. High-confidence regulatory networks (A, B, C levels) from the Dorothea database were loaded, and the TF activity score for each cell was calculated using the Viper algorithm. After normalization, PCA, and T-distributed Stochastic Neighbor Embedding dimensionality reduction, cells were clustered. The average TF activity was then calculated for different risk groups, and significantly altered TFs were screened. Heatmaps were used to display the activity patterns of these TFs in high-risk and low-risk groups.

### Statistical analysis

This study employed various statistical analysis methods, integrating MR techniques to evaluate the causal relationship between lipid traits and the risk of AS. Initially, univariate and multivariable MR analyses were performed using IVW regression and MR-Egger regression to determine the causal effects between lipid traits and AS. Sensitivity analyses were performed, including heterogeneity tests, horizontal pleiotropy tests, and MR-PRESSO outlier detection. To account for errors introduced by multiple testing, we applied false discovery rate correction to adjust the results of each MR analysis. The q-value correction method was used, with a q-value threshold < 0.1 set to identify significant associations. Results with *P*-values < 0.05, even if the *q*-value > 0.1, were considered as potential suggestive evidence. Additionally, drug target MR analysis identified lipid-lowering drug targets from the DrugBank database. The causal associations between these target genes and AS risk were then assessed. Combining colocalization analysis and SMR analysis, we explored shared causal variants between lipid traits and AS risk. For the analysis of single-cell RNA-seq data, we utilized R packages such as Seurat and hdWGCNA for cell type identification, gene module analysis, and GSEA. TF activity was parsed using the Dorothea and Viper tools. All statistical analyses were performed in the R 4.2.2 environment using packages like “TwoSampleMR” for data analysis.

## Results

### Causal relationship between lipid traits and AS

**Figure 1.**
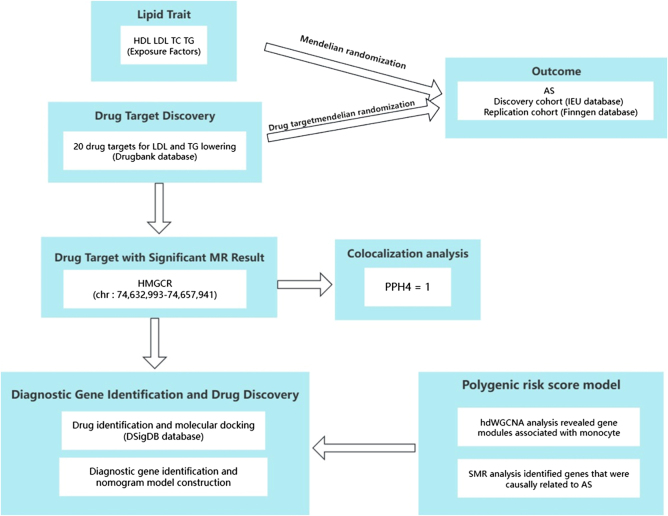
Schematic of the experimental workflow in this study.

**Figure 2.**
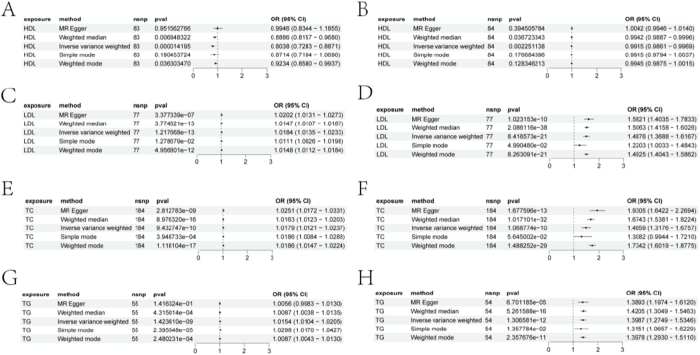
Associations between lipid traits and ASCVD risk. A, C, E, and G show forest plots depicting the relationship between HDL, LDL, TC, and TG and ASCVD risk in the discovery cohort. B, D, F, and H show forest plots of the same lipid traits in the replication cohort. Data are presented as odds ratios (ORs) with 95% confidence intervals (error bars). ORs less than 1.00 indicate a reduced risk of atherosclerosis.

Figure 1 provides a technical roadmap of the study. Figure 1 shows the results of univariate MR analysis assessing the causal relationships between four lipid traits and AS in the discovery and replication cohorts. In the discovery cohort, genetically predicted decreases in HDL levels were significantly associated with an increased risk of AS (OR = 0.8038, 95% CI 0.7283-0.8871, *P* = 0.000014) using the weighted median method. This association was also confirmed using the IVW method (OR = 0.8886, 95% CI 0.8157-0.9680, *P* = 0.0068). Similarly, simple and weighted mode methods showed consistent results (Fig. 2A). In the replication cohort, genetically predicted changes in HDL levels also showed a significantly associated with health outcomes (Fig. 2B). Detailed information on the integration of instrumental variables, HDL, and outcomes is provided in Table 1 and Supplemental Digital Content Tables S1-S4, available at: http://links.lww.com/JS9/E859.

Genetically predicted increases in LDL levels were significantly associated with an increased risk of AS (MR Egger method: OR = 1.0202, 95% CI 1.0131-1.0273, *P* = 3.38e-07; weighted median method: OR = 1.0147, 95% CI 1.0107-1.0188, *P* = 9.95e-13; IVW method: OR = 1.0184, 95% CI 1.0135-1.0233, *P* = 1.22e-13; simple mode method: OR = 1.0111, 95% CI 1.0034-1.0190, *P* = 6.27e-03; weighted mode method: OR = 1.0148, 95% CI 1.0113-1.0183, *P* = 2.10e-12). These results consistently indicate that genetically predicted increases in LDL levels are significantly associated with an increased risk of AS, further emphasizing the importance of LDL in the development of cardiovascular diseases (Fig. 2C). In the replication cohort, genetically predicted increases in LDL levels also showed a significant trend toward increased AS risk (Fig. 2D). Detailed information on the integration of instrumental variables, LDL, and outcomes is provided in Supplemental Digital Content Tables S5-S8, available at: http://links.lww.com/JS9/E859.

Genetically predicted increases in TC levels were significantly associated with an increased risk of AS (MR Egger method: OR = 1.0251, 95% CI 1.0172-1.0331, *P* = 2.81e-09; weighted median method: OR = 1.0163, 95% CI 1.0123-1.0203, *P* = 8.98e-16; IVW method: OR = 1.0179, 95% CI 1.0121-1.0237, *P* = 9.43e-10; simple mode method: OR = 1.0186, 95% CI 1.0084-1.0288, *P* = 3.95e-04; weighted mode method: OR = 1.0186, 95% CI 1.0147-1.0224, *P* = 1.12e-17) (Fig. 2E). These results consistently indicate that genetically predicted increases in TC levels are significantly associated with an increased risk of AS, further emphasizing the importance of TC in the development of cardiovascular diseases. In the replication cohort, genetically predicted increases in TC levels similarly showed a significant trend toward an increased risk of AS (Fig. 2F). Detailed information on integrating instrumental variables, TC, and outcomes is in Supplemental Digital Content Tables S9-S12, available at: http://links.lww.com/JS9/E859.

Genetically predicted increases in TG levels were also significantly associated with an increased risk of AS (MR Egger method: OR = 1.0056, 95% CI 0.9983-1.0130, *P* = 1.42e-01; weighted median method: OR = 1.0087, 95% CI 1.0038-1.0135, *P* = 4.32e-04; IVW method: OR = 1.0154, 95% CI 1.0104-1.0205, *P* = 1.42e-09; simple mode method: OR = 1.0298, 95% CI 1.0170-1.0427, *P* = 2.40e-05; weighted mode method: OR = 1.0087, 95% CI 1.0043-1.0130, *P* = 2.48e-04) (Fig. 2G). In the replication cohort, genetically predicted increases in TG levels similarly showed a significant trend toward an increased risk of AS (Fig. 2H). These results consistently indicate that genetically predicted increases in TG levels are significantly associated with an increased risk of AS, further emphasizing the importance of TG in the development of cardiovascular diseases. Detailed information on integrating instrumental variables, TG, and outcomes can be found in Supplemental Digital Content Tables S13-S16, available at: http://links.lww.com/JS9/E859.

### Multivariable MR analysis of lipid traits and AS

**Figure 3.**
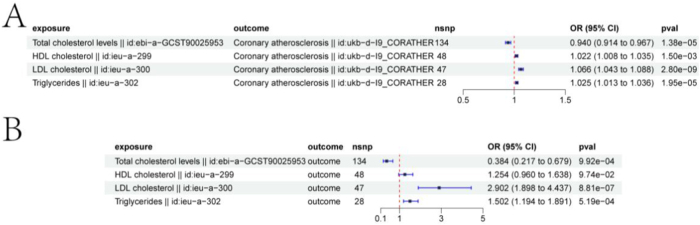
Results of exploring the association between lipid traits and ASCVD risk using multivariable Mendelian randomization (MVMR). Panels A and B show forest plots of the relationship between lipid traits and ASCVD risk in the discovery and replication cohorts, respectively. Data are presented as odds ratios (ORs) with 95% confidence intervals (error bars). ORs less than 1.00 indicate a reduced risk of atherosclerosis.

**Figure 4.**
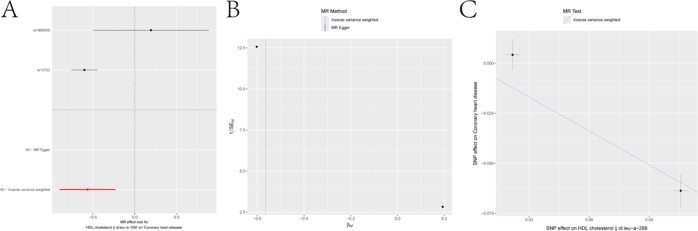
MR analysis of drug targets and ASCVD risk. Panel A shows a forest plot of the association between drug targets and ASCVD risk. Panel B depicts the causality of HMGCR on atherosclerosis. Panel C shows the effect of SNPs on the outcome across different models, where the slope of the line represents the causal relationship.

The relationship between each lipid trait and AS outcomes was assessed using MVMR analysis, with shared SNPs among exposures excluded. Consistent results were observed in both the discovery cohort (Fig. 3A) and the replication cohort (Fig. 3B). TC levels were significantly negatively associated with coronary ASCVD in cohorts (Discovery cohort: OR = 0.940, 95% CI = 0.914–0.967, *P* = 1.38e−05; Replication cohort: OR = 0.384, 95% CI = 0.217–0.679, *P* = 9.92e−04). Additionally, LDL and TG were significantly positively associated with coronary ASCVD in both cohorts, with more pronounced associations in the replication cohort (LDL: OR = 2.902, 95% CI = 1.898–4.437, *P* = 8.81e−07; TG: OR = 1.502, 95% CI = 1.194–1.891, *P* = 5.19e−04). HDL showed a slight positive association in the discovery cohort (OR = 1.022, 95% CI = 1.008–1.035, *P* = 1.50e−03), but this association did not reach statistical significance in the replication cohort (OR = 1.254, 95% CI = 0.960–1.638, *P* = 9.74e−02) (Figure 4A-C).

Table 2 presents the associations between lipid traits and AS estimated using MVMR. Overall, significant causal effects of lipid traits on AS were identified, aligning with previous observations.

**Table 1:** Description of traits used in MR analyses

Traits	Data source	Data ID	Sample size	Number of SNPs
ieu-a-299	IEU database	ieu-a-299	187 167	2 447 442
IEU database	IEU database	ieu-a-300	173 082	2 437 752
IEU database	IEU database	ieu-a-302	177 861	2 439 433
IEU database	IEU database	ebi-a-GCST90025953	437 878	4 232 052
Finngen database	Finngen database	summary_stats_finngen_R11_I9_CORATHER	56 685	378 019
ukb-d-I9_CORATHER	IEU database	ukb-d-I9_CORATHER	361 194	13 586 589

**Table 2:** Associations between lipid traits and ASCVD estimated using MVMR

Exposure	Outcome	Cochran *Q* test (discovery queue)		Cochran *Q* test (replicated queue)	
		*Q* value	*P*	*Q* value	*P*
HDL	Atherosclerosis	524.0764	1.55E-40	888.9843	7.92E-102
LDL					
TG					
TC					

### Primary lipid-modulating effects and AS risk of genetically predicted drug targets

We collected instrumental variables for lipid-modifying drug targets, with detailed information provided in the Supplementary Digital Content material, available at: http://links.lww.com/JS9/E859. HMGCR was identified as a drug target associated with a reduced risk of AS. This finding not only underscores the effectiveness of the genetic instruments but also indicates a high instrument strength, with F-statistics exceeding the threshold of 10. The results of heterogeneity, drug target MR analysis, and horizontal pleiotropy testing are presented in Supplementary Digital Content Tables S17-S19, available at: http://links.lww.com/JS9/E859.

### Drug target colocalization and molecular docking

**Figure 5.**
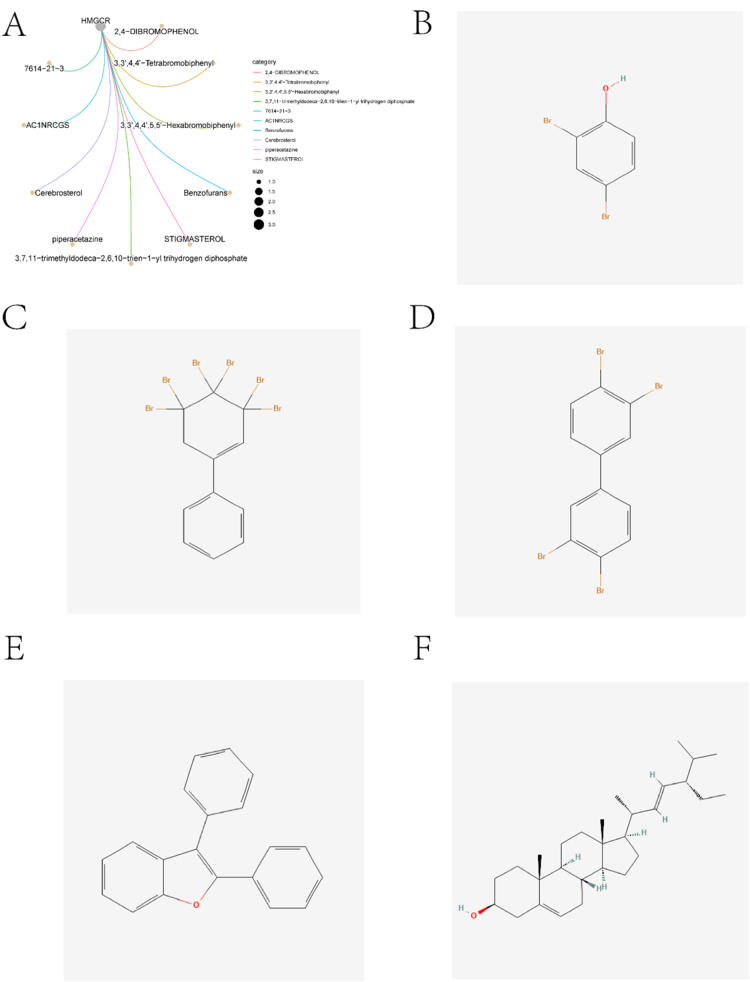
The drug identification. A shows the results of the drug enrichment analysis. B-F is the 2D chemical structures of the following compounds: 2,4-Dibromophenol, 3,3’,4,4’-Tetrabromobiphenyl, 3,3’,4,4’,5,5’-Hexabromobiphenyl, Benzofurans, and Stigmasterol.

**Figure 6.**
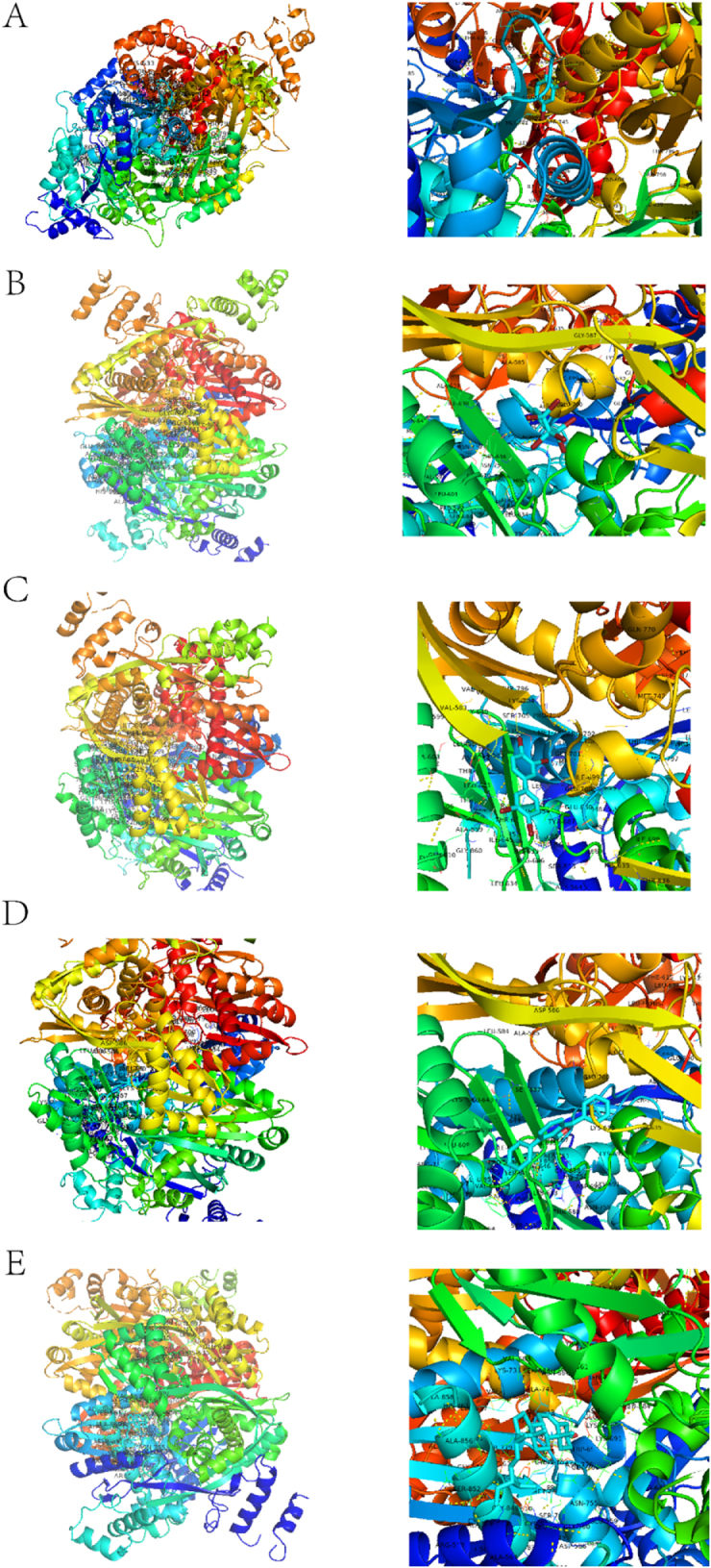
Molecular docking results. A-E show the molecular docking results of HMGCR with 2,4-Dibromophenol, 3,3’,4,4’-Tetrabromobiphenyl, 3,3’,4,4’,5,5’-Hexabromobiphenyl, Benzofurans, and Stigmasterol, respectively.

Given the high credibility of the causal variants linking HMGCR expression levels to ASCVD risk, colocalization analysis (PPH4 = 1) indicated that linkage disequilibrium is not a confounding factor for the MR findings observed for this gene (Supplemental Digital Content Tables S20, available at: http://links.lww.com/JS9/E859). Drug enrichment analysis revealed significant associations between HMGCR and 2,4-Dibromophenol, 3,3’,4,4’-Tetrabromobiphenyl, 3,3’,4,4’,5,5’-Hexabromobiphenyl, Benzofurans, and Stigmasterol. The 2D structures of these five compounds are shown in Figure 5. We performed molecular docking analysis between these five drugs and HMGCR. Binding energies less than −4.25 kcal · mol^−1^ indicate moderate binding activity. Energies below −5.0 kcal · mol^−1^ suggest good binding activity. Energies under −7.0 kcal · mol^−1^ represent strong binding activity. Figure 6 shows the docking results of HMGCR with 2,4-Dibromophenol (−5.6 kcal/mol), 3,3,’4,4’-Tetrabromobiphenyl (−6.6 kcal/mol), 3,3’,4,4’,5,5’-Hexabromobiphenyl (−6.1 kcal/mol), Benzofurans (−7.3 kcal/mol), and Stigmasterol (−7.7 kcal/mol). HMGCR demonstrated stronger binding with Stigmasterol and Benzofurans.

### Construction of a polygenic score model for AS

**Figure 7.**
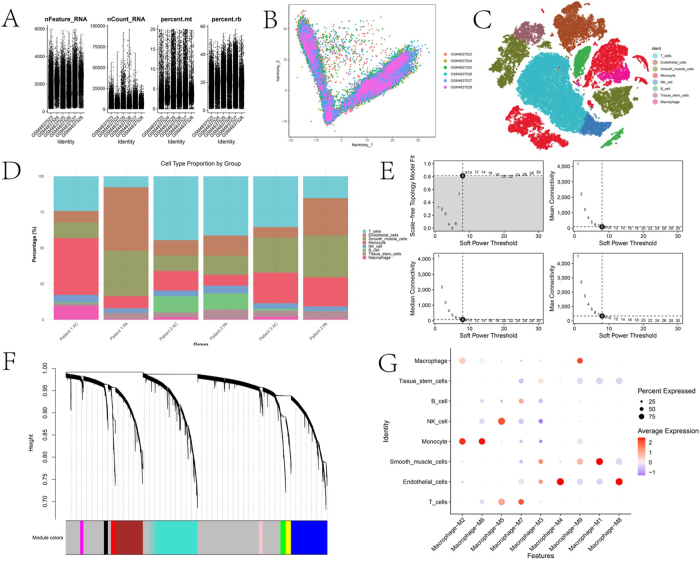
Basic analysis of scRNA-seq data and identification of key module genes. A shows the distribution of nFeature_RNA, nCount_RNA, percent.Mt, and percent.Rb across different samples, indicating good data quality with mitochondrial and ribosomal gene proportions within reasonable ranges. B shows the distribution of different samples after Harmony integration analysis. C displays the t-SNE visualization after Harmony integration, clearly distinguishing major cell types, including T cells, macrophages, endothelial cells, and smooth muscle cells. D illustrates the differential proportions of cell types across different groups, showing significant enrichment of macrophages and smooth muscle cells in the AS group. E presents the soft threshold selection results from WGCNA analysis, showing that a soft threshold of 6 satisfies the scale-free property and has good connectivity. F shows the hierarchical clustering of gene modules based on WGCNA, demonstrating that modules correlate with specific cell types. G displays a heatmap of the expression patterns of different gene modules in major cell types, highlighting the unique features of the macrophage and T cell modules.

In this study, we analyzed single-cell sequencing data to evaluate cell characteristics and quality control metrics of the samples. The median distributions of nFeature_RNA and nCount_RNA were consistent across samples. Additionally, the proportions of mitochondrial (percent.mt) and ribosomal (percent.rb) genes were within reasonable ranges (Fig. 7A). Batch effects were successfully removed using the Harmony embedding method (Fig. 7B), achieving apparent clustering of primary cell types such as T cells, macrophages, endothelial cells, and smooth muscle cells (Fig. 7C). Cell proportion analysis showed significant differences in cell types between the AS and control groups. Macrophages and smooth muscle cells were significantly enriched in the AS group (Fig. 7D). Based on Weighted Gene Co-expression Network Analysis (WGCNA) soft-thresholding screening, a soft threshold of 6 was chosen to maintain scale-free network properties while ensuring good network connectivity. Hierarchical clustering analysis of gene modules identified specific modules associated with cell types (Fig. 7E-F). The gene lists for different modules are provided in Supplemental Digital Content Tables S21, available at: http://links.lww.com/JS9/E859. Module gene expression heatmaps further validated the specific expression patterns of each module. The monocyte cell cluster showed significant correlations with the M2 and M6 gene modules (Fig. 7G).

**Figure 8.**
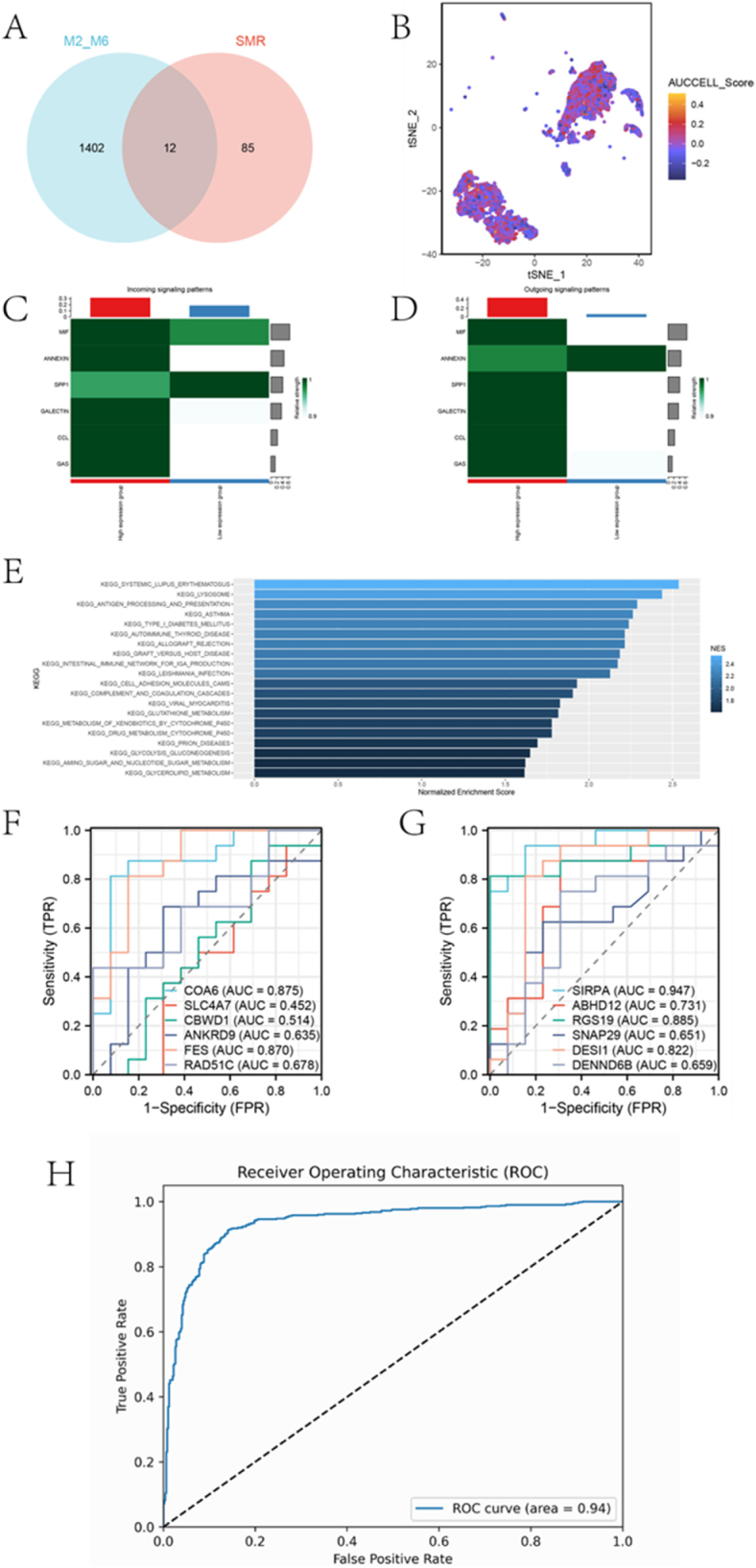
Construction and validation of the polygenic risk score model. A shows a Venn diagram illustrating the intersection of module genes and causal relationships identified by SMR analysis. B displays a t-SNE plot showing the distribution of AUCCELL scores in single-cell data, evaluating the scoring distribution in the macrophage population. C and D present signaling pathway analysis, indicating significant differences in the incoming and outgoing signal strengths of signaling molecules such as MIF, ANNEXIN, SPP1, and CCL between high and low-expression groups. E shows the results of GSEA analysis between high and low expression groups. F presents GSVA analysis, revealing significantly activated pathways between high and low-expression cell populations. G and H show ROC curve analysis, evaluating the diagnostic efficiency of candidate biomarkers, with Panel H depicting the ROC curve based on hub genes.

On the other hand, SMR analysis identified 97 genes causally associated with AS (Supplemental Digital Content Tables S22, available at: http://links.lww.com/JS9/E859). Intersecting these genes with those in the M2 and M6 modules yielded 12 overlapping genes (Fig. 8A). The AUCell algorithm was used to score the signature composed of these 12 genes in the monocyte cell cluster (Fig. 8B). Cell communication analysis between the two groups of cells showed significant differences in signaling molecules such as macrophage migration inhibitory factor (MIF), Annexin A1, secreted phosphoprotein 1 (SPP1), and chemokine (C-C motif) ligand 2 (CCL2). MIF and Annexin A1 exhibited stronger outgoing signal intensities in the high-expression group, highlighting their essential roles in microenvironment regulation (Fig. 8C-D). Gene Set Variation Analysis (GSVA) revealed molecular signatures between the two groups of cells. Multiple pathways, including glycerolipid metabolism, glycolysis/gluconeogenesis, glutathione metabolism, and amino sugar and nucleotide sugar metabolism, were significantly activated.

Additionally, the active complement and coagulation cascades, as well as viral myocarditis-related immune pathways, further emphasized the synergistic effects between metabolism and immunity (Fig. 8E). Receiver Operation Characteristic (ROC) curve analysis identified several potential biomarkers, including cytochrome c oxidase assembly factor 6 (COA6, the area under the curve (AUC) = 0.875), feline sarcoma oncogene (FES, AUC = 0.870), and signal-regulatory protein alpha (SIRPA, AUC = 0.947), which demonstrated high sensitivity and specificity in disease diagnosis (Fig. 8F-G). A logistic regression model was built using a 12-gene signature. It achieved an AUC of 0.94 for predicting AS, indicating high diagnostic accuracy (Fig. 8H).

## Discussion

This study systematically investigated the causal relationship between lipid traits and the risk of ASCVD using univariate MR analysis in discovery and validation cohorts. The results indicated that decreased levels of HDL and elevated levels of LDL, TC, and TG significantly increased the risk of AS, confirming the critical role of these lipid traits in cardiovascular health^[19–21]^. Among them, LDL was confirmed to be an especially potent pathogenic factor, further highlighting its central position in the pathogenesis of AS^[22,23]^. These findings not only highlight the importance of lipid-regulating therapies for ASCVD prevention, but also suggest potential interventions targeting HDL and TG for better cardiovascular risk management. Sensitivity analyses were conducted to minimize pleiotropic bias and ensure result reliability. However, limitations such as residual confounding, population differences, and unexplored gene-environment interactions remain. These issues need further investigation in future studies.

This study explored the causal relationship between lipid traits and coronary AS using MVMR methods. The results demonstrated that TC levels were significantly negatively associated with ASCVD. In contrast, LDL and TG levels were significantly positively associated with ASCVD, with the associations for LDL and TG being more pronounced in the replication cohort. HDL levels were negatively associated with ASCVD in the discovery cohort, suggesting a potential protective role for HDL; however, this association did not reach statistical significance in the replication cohort. Notably, MVMR adjusted for collinearity among lipid traits and excluded shared SNPs. This resulted in independent estimates of the causal effects of TC. These estimates differed from those observed in univariable MR analyses. This discrepancy may reflect that the positive causal effects of LDL and TG on ASCVD were removed in the MVMR analysis, highlighting the negative association of TC, primarily driven by the protective effect of HDL. This study showed that lipid traits have significant causal effects on ASCVD. The trends were similar to previous UVMR findings, but the MVMR results offered more accurate insights into the independent effects of each lipid trait.

HMGCR was identified as a potential drug target associated with a reduced risk of ASCVD through genetic target prediction analysis. Our results showed a significant causal relationship between HMGCR genetic instruments and AS risk^[24,25]^. The reliability of HMGCR as a drug target was further supported by the findings from heterogeneity, drug-target MR analysis, and horizontal pleiotropy analysis. Additionally, colocalization analysis (PPH4 = 1) indicated that linkage disequilibrium was not a confounding factor for the MR findings of HMGCR, thereby validating the credibility of its causal role. Molecular docking results demonstrated strong binding between HMGCR and Stigmasterol as well as Benzofurans, suggesting that HMGCR could be a potential drug target for AS. Cholesterol levels are closely associated with the development of AS, and studies have shown that HMGCR plays a central role in cholesterol synthesis^[26]^. HMGCR is the therapeutic target of commonly used AS medications, such as statins. Man K. S. Lee *et al* identified the AMPK-HMGCR axis-related signaling pathways as involved in regulating cholesterol homeostasis in hematopoietic stem and progenitor cells in an AS mouse model. Inhibition of this regulatory mechanism accelerates the onset and progression of AS^[27]^.

On the other hand, this article systematically analyzed single-cell RNA sequencing (scRNA-seq) data, comprehensively revealing the cellular composition, gene networks, and molecular mechanisms underlying ASCVD. It clearly identified major cell types, including T cells, macrophages, endothelial cells, and smooth muscle cells. Based on WGCNA, two modules, M2 and M6, were identified as being significantly associated with monocytes, and the functional characteristics of genes within these modules were further confirmed through AUCell scoring. Additionally, using SMR analysis, 97 genes causally related to AS were identified, 12 of which (COA6, SLC4A7, CBWD1, ANKRD9, FES, RAD51C, SIRPA, ABHD12, RGS19, SNAP29, DESI1, and DENND6B) overlapped with the aforementioned modules, indicating their importance in monocyte function and the progression of AS. Chen *et al* also identified COA6 as a gene relevant to diagnosing AS through bioinformatics and machine learning analyses of copper poisoning-related genes in AS^[28]^. SLC4A7, a key sodium and bicarbonate transporter, has been reported to potentially be dysregulated during the process of AS^[29]^. Elisavet Karamanavi *et al* reported that FES deficiency leads to larger AS plaques with more monocytes^[30]^. Li *et al* found that CRISPR/Cas9-mediated knockdown of RGS19 and KPTN in human hepatocyte cell lines resulted in decreased secretion of APOB100 and lipids into the culture medium^[31]^.

Inter-cellular communication analysis revealed that signals such as MIF, ANNEXIN, and SPP1 exhibit stronger outgoing patterns in the high-expression group of monocytes, while CCL signals show significant differences across different expression groups. Sinitski *et al* reviewed blocking chemokines as a promising anti-atherosclerotic strategy to coordinate leukocyte recruitment in AS^[32]^. GSVA further identified active metabolic and immune-related pathways, highlighting the synergy between the two. The analysis showed that multiple metabolic pathways were significantly activated, including glycolysis/gluconeogenesis and glutathione metabolism. These metabolic pathways are closely associated with inflammation and immune responses, potentially regulating cell survival and function during the pathological process. Glycolysis provides energy and intermediates required for vital activities of the organism, and abnormal increases in its flux accelerate the progression of AS^[33]^. Oren Rom *et al* found that glycine-based therapy-induced glutathione biosynthesis can alleviate AS^[34]^. Furthermore, lysosomal compartment dysfunction plays a central role in the etiology and pathogenesis of AS^[35]^. ROC curve analysis demonstrated that genes such as COA6, FES, and SIRPA exhibit high diagnostic value. A predictive model constructed based on 12 genes achieved an AUC of 0.94, indicating excellent diagnostic performance. The comprehensive analysis of this study not only elucidates the cellular heterogeneity, metabolic, and immune characteristics of ASCVD but also proposes potential molecular markers and diagnostic models, offering new directions for understanding the pathogenesis of AS and improving clinical diagnosis.

However, the limitations of this study include the dependence of MVMR analysis on the validity and independence of SNPs, as well as the potential impact of heterogeneity across different populations on the generalizability of the results. Future research should further investigate the differential roles of lipid metabolic pathways and consider other potential regulatory factors to gain a more comprehensive understanding of the relationship between lipid traits and AS. Additionally, the lack of independent cohort validation and spatial transcriptomics data means that these findings should be further verified in larger-scale, multi-center datasets to more comprehensively uncover the molecular mechanisms of the disease and promote its clinical translation.

## Conclusion

Our study provides robust evidence supporting the causal relationship between lipid traits and AS. The genetic predisposition to higher LDL, TC, and TG levels significantly increases AS risk, emphasizing the importance of lipid metabolism in ASCVD progression. The identification of HMGCR as a potential drug target, along with its strong interaction with specific compounds, opens avenues for targeted therapeutic interventions. Furthermore, the integration of multi-gene risk scores and molecular mechanisms paves the way for future research into personalized prevention and treatment strategies for AS. These findings underscore the relevance of genetic insights in understanding and managing AS and related cardiovascular conditions.

## Data Availability

The data used in this paper are from the GEO database (https://www.ncbi.nlm.nih.gov/geo/), IEU database (https://gwas.mrcieu.ac.uk/), and Finngen database (https://www.finngen.fi/en/access_results).
